# Recent Advances in Signal Amplification to Improve Electrochemical Biosensing for Infectious Diseases

**DOI:** 10.3389/fchem.2022.911678

**Published:** 2022-06-13

**Authors:** Xingcheng Zhou, Daena A. Schuh, Lauren M. Castle, Ariel L. Furst

**Affiliations:** ^1^ Department of Chemical Engineering, Massachusetts Institute of Technology, Cambridge, MA, United States; ^2^ Center for Environmental Health Sciences, Massachusetts Institute of Technology, Cambridge, MA, United States

**Keywords:** biosensors, electrochemistry, signal amplification, infectious diseases, diagnostics, healthcare, point-of-care (POC)

## Abstract

The field of infectious disease diagnostics is burdened by inequality in access to healthcare resources. In particular, “point-of-care” (POC) diagnostics that can be utilized in non-laboratory, sub-optimal environments are appealing for disease control with limited resources. Electrochemical biosensors, which combine biorecognition elements with electrochemical readout to enable sensitive and specific sensing using inexpensive, simple equipment, are a major area of research for the development of POC diagnostics. To improve the limit of detection (LOD) and selectivity, signal amplification strategies have been applied towards these sensors. In this perspective, we review recent advances in electrochemical biosensor signal amplification strategies for infectious disease diagnostics, specifically biosensors for nucleic acids and pathogenic microbes. We classify these strategies into target-based amplification and signal-based amplification. Target-based amplification strategies improve the LOD by increasing the number of detectable analytes, while signal-based amplification strategies increase the detectable signal by modifying the transducer system and keep the number of targets static. Finally, we argue that signal amplification strategies should be designed with application location and disease target in mind, and that the resources required to produce and operate the sensor should reflect its proposed application, especially when the platform is designed to be utilized in low-resource settings. We anticipate that, based on current technologies to diagnose infectious diseases, incorporating signal-based amplification strategies will enable electrochemical POC devices to be deployed for illnesses in a wide variety of settings.

## Introduction

Infectious disease is at the forefront of the global consciousness due to the ongoing COVID-19 pandemic. Understanding the global burden of such diseases is necessary to both improve population-wide outcomes and treat patients on an individual level. A key barrier to gaining this knowledge is inequality in access to healthcare resources, especially diagnostics. Thus, significant effort has been devoted to the development of technologies to improve equity in diagnostics availability, especially in low-resource settings. One major area of research for the development of such platforms is electrochemical biosensors, which enable quantitative target readout using inexpensive, simple equipment.

Electrochemical biosensors combine biorecognition elements, or biomolecules that specifically recognize and bind an analyte, with electrochemical readout to enable sensitive and specific sensing. The binding of an analyte induces a concentration-dependent change in the system, such as resistance or capacitance, that can be detected through a signal processer. (Hernandez-Vargas et al., 2018). The gold standard electrochemical device is the glucose meter, which fundamentally improved patient outcomes and quality of life. The glucometer is considered a “point-of-care” (POC) sensor because it can be used in non-laboratory, sub-optimal environments without requiring specialized equipment or training (Gubala et al., 2012; Nayak et al., 2017). Notably, the sensor utilizes inherent signal amplification through the activity of the glucose oxidase enzymes, which catalyzes the conversion of glucose to gluconic acid. The reduced form of the enzyme reduces an electroactive mediator, which is then oxidized at the electrode surface, generating a detectable signal. This mediator is cycled through the oxidized and reduced forms, improving the signal. Signal amplification strategies, not just limited to usage of biological materials like enzymes, are essential for both laboratory and POC diagnostics, as this generally improves the precision and accuracy of the device while also lowering the limit of detection (LOD).

**FIGURE 1:**
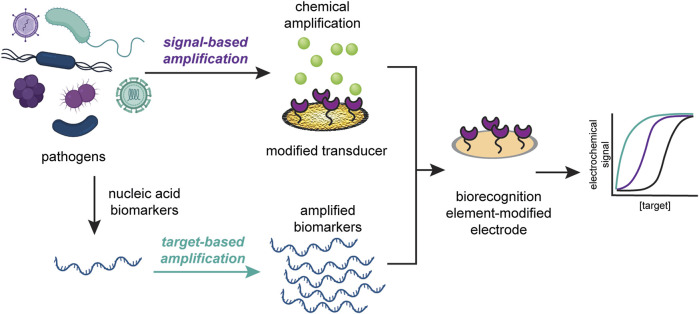
Signal amplification strategies can be classified into target-based amplification (top) or signal-based amplification (bottom). Target-based strategies increase the copies of detectable target, while signal-based strategies modify the transducer to increase the detectable signal.

Here, we discuss the recent advances in the improvement of electrochemical sensors for infectious disease diagnosis through signal amplification strategies. These strategies can be divided into two classes: target-based amplification and signal-based amplification. We define target-based amplification as methods that increase the number of detectable targets and signal-based amplification as methods that modify the transducer to increase the detectable signal. We then highlight recent improvements in signal amplification strategies for two types of instigators of infectious disease: viruses and pathogenic microbes, as seen in Figure 1. We further argue that signal amplification strategies should be designed with application location and disease target in mind, and that the resources required to produce and operate the sensor should reflect the its proposed application. We anticipate that, based on current technologies to diagnose infectious diseases, incorporating signal-based amplification strategies will enable electrochemical POC devices to be deployed for such illnesses in a wide variety of settings.

### Target-based amplification strategies

**FIGURE 2:**
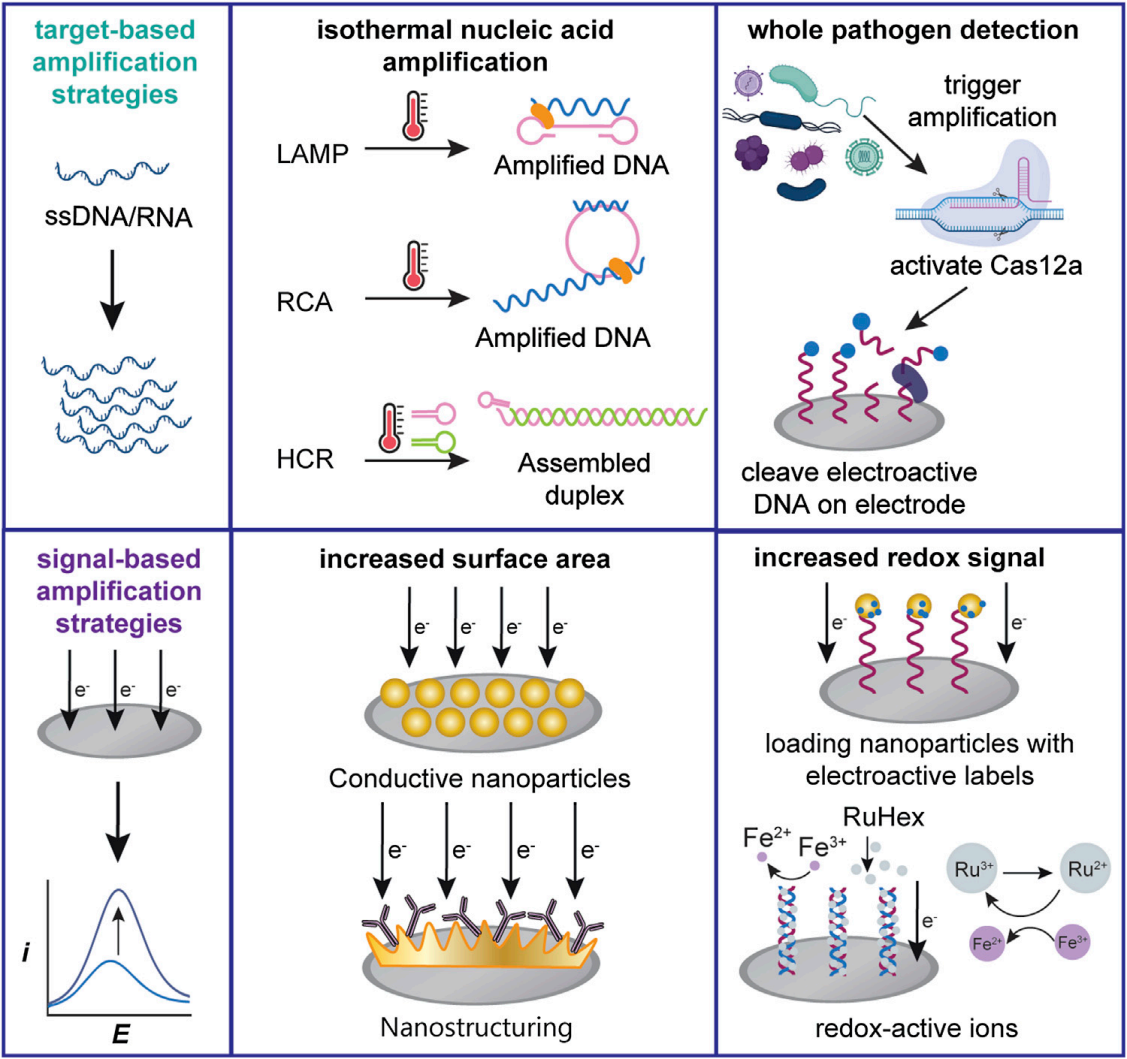
Schemes of several commonly used electrochemical signal amplification strategies. Target-based amplification strategies can be applied towards both nucleic acid or cellular targets. Signal-based strategies generally increase the transducer surface area or detectable redox signal.

Target-based amplification strategies increase the abundance of the targeted analyte and are commonly used for the detection of infectious disease nucleic acid sequences. DNA amplification through the real-time polymerase chain reaction (qPCR) with quantitative detection, usually through fluorescence or colorimetric monitoring, is the most prevalent method for many of these diagnostics (Valasek and Repa, 2005; Mackay et al., 2002; Watzinger et al., 2006). However, qPCR-capable thermocyclers are costly, with price tags of up to $120,000. Thus, detection without necessitating fluorescent readout is of great interest. To that end, qPCR has been integrated with electrochemical detection where the target DNA is quantified during amplification, commonly through the use of intercalating redox probes such as methylene blue (MB) (Kelley et al., 1997; Erdem et al., 2000; Erdem et al., 2001). Despite these advances, the equipment needed for the thermal amplification remains prohibitively costly and unsuitable for non-laboratory settings (Mori et al., 2001). In comparison, isothermal nucleic acid amplification strategies, such as loop-mediated isothermal amplification (LAMP) and rolling circle amplification (RCA), as seen in Figure 2, are promising alternatives for nucleic acid amplification that reduce the need for a thermocycler and are faster than qPCR (Soliman et al. 2009; Haible et al. 2006), making them a more attractive option for POC diagnostics.

Generally, LAMP involves a series of primers that recognize different regions of the target sequence. The application of a specific DNA polymerase combined with primers forms loops, enabling additional amplification (Notomi et al., 2000). Nucleic acid products generated from LAMP can be detected electrochemically through the use of intercalating probes, (Safavieh et al., 2014; Xie et al., 2014; Martin et al., 2016), and this method has been quickly adapted for the detection of SARS-CoV-2 during the COVID-19 pandemic. In one example, a sensor developed by Ramírez-Chavarría et al. detects SARS-CoV-2 from wastewater samples using screen-printed electrodes with reverse-transcription LAMP (Ramírez-Chavarría et al., 2022). Viral genetic material was extracted and concentrated with an electronegative membrane, amplified with LAMP, and electrochemically detected with MB as the intercalating agent. An LOD of 38 × 10^−6^ ng/ul was reported for the sensor. Minimal instrumentation was used and the sensor can be integrated into a single device, which is ideal for POC automated analysis.

One disadvantage of LAMP is that random amplification can lead to false positive readings, so it is often combined with secondary detection techniques to remove false positive signals (Kaminski et al., 2021). In one clever example, Zamani et al. combined LAMP amplification of viral genetic material with CRISPR-Cas12a endonuclease activation and electrochemical readout (Zamani et al., 2021a). This workflow was used to detect human papillomavirus (HPV) from clinical samples. Following HPV DNA isolation from cervical swabs and amplification with LAMP, the Cas12a enzyme was activated by the amplicons. This enzyme cleaves single-stranded DNA after activation, in this case MB-coupled DNA immobilized on gold electrodes, reducing the electrochemical signal. The LOD of the assay was 1.2 × 10^4^ copies/ml of HPV, a clinically relevant concentration. Furthermore, the sensor platform itself was developed for low-resource setting applications; the electrode was constructed from gold leaf without requiring equipment or a clean room. This method significantly decreased the fabrication cost of the electrodes (Zamani et al., 2021b).

RCA is another isothermal nucleic acid amplification method that enables facile target amplification: single-stranded nucleic acids are amplified from a short primer annealed to a circular template (Ali et al., 2014). This amplification strategy has also been applied in combination with CRISPR-Cas12a for the electrochemical detection of nucleic acids, including the DNA of parvovirus B19 (Qing et al., 2021). In this assay, the electrode was pre-blocked with nucleic acid probes. In the presence of the target, RCA produced nucleic acids that activate the Cas12a enzyme to initiate ssDNA cleavage. The enzyme then digests the electrode-blocking strand and allows a MB-labeled probe to adsorb to the electrode, thus generating an electrochemical signal. The LOD for parvovirus DNA was 0.52 aM, with a detection range from 50 aM to 10 pM. This method can be reprogrammed for the universal detection of any nucleic acid and is also free of any immobilization steps, significantly simplifying the preparation steps.

One significant disadvantage of both LAMP and RCA is that both methods require enzymes, which can increase the stringency of storage requirements for assays and the cost-per-test. In contrast, an isothermal amplification method that does not require enzymes for activity, termed the hybridization chain reaction (HCR) (Evanko, 2004), has also been applied to electrochemical detection of amplified nucleic acids. In HCR, an initiator oligonucleotide triggers the self-assembly of duplexed DNA from DNA hairpins. We note that in recent advances, electrochemical HCR has mostly been used to detect microRNAs (miRNAs), but we anticipate this platform to be readily adaptable to the sensing of infectious disease biomarkers (Zhou et al., 2019; Guo et al., 2020; Miao and Tang, 2020).

Combinations of multiple nucleic acid amplification strategies have also been developed. A combination of HCR, RCA, and dual DNA walkers was applied by Zhang et al. towards sensing the nucleic acids from the pathogen *Escherichia coli* O157:H7 (Zhang et al., 2020). However, such complexity is often undesirable for low-resource settings because of multi-step requirements and potential storage limitations. In summary, one-pot isothermal amplification methods have been applied to electrochemical sensing of nucleic acids in infectious diseases with great success, and these strategies can be fused with other diagnostic methods to improve detection.

While nucleic acid markers can easily be amplified and detected through hybridization with capture probes, one limitation for nucleic acid sensing is that isolating genetic material from whole pathogens requires lysing, purification, and isolation. Therefore, it is advantageous to directly detect whole pathogens from clinical samples. In this section, we focus on the detection of whole cells. However, target amplification of whole cells is challenging as there are no fast, reliable techniques to create new copies of cells *in situ*, other than directly culturing from patient samples, which can take several days (Klass et al., 2021). To circumvent these challenges, nucleic acids are often used as proxies for amplification of non-nucleic-acid targets or as intermediates to trigger signal generation. Because of their ease of incorporation, the isothermal nucleic acid amplification strategies described in the previous section have also be applied to whole cell detection.

One example of a nucleic acid amplification sensor design for whole cells was generated by Liu et al. for the detection of the foodborne bacterial pathogen *Salmonella typhimurium* (Liu et al., 2022). The sensor was based on a combination of aptamers, HCR, and CRISPR-Cas12a. Aptamers are short, single stranded nucleic acid molecules that can bind to a target molecule. *S. typhimurium* aptamers were first hybridized to an immobilized HCR binder. In the presence of target, the aptamer binds to the cell instead, exposing the HCR binding site and triggering HCR. The product then activates Cas12a, cleaving MB-modified nucleic acids on an electrode surface, leading to a decrease in the electrochemical signal. The LOD was 20 CFU/ml, and the dynamic range was 10–10^8^ CFU/ml. This method stands out, as it minimizes the use of enzymes during amplification and was capable of identifying specific bacterial strains.

One key advantage of RCA as compared to LAMP is that it can be conducted on a solid surface (Konry et al., 2011), including on cell surfaces, making it ideal for whole cell detection. However, electrochemical RCA has mostly been used for sensing nucleic acids, proteins, and small molecules (Fan et al., 2018; Ciftci et al., 2020; He et al., 2020). In two examples of whole cell detection, this method was applied to electrochemically detect circulating tumor cells rather than pathogenic microbes (Sun et al., 2018; Shen et al., 2019). We believe that, given the unique advantages of RCA, it has potential for the sensitive detection of infectious diseases.

In addition to the aforementioned methods, other isothermal nucleic acid amplification techniques such as strand-displacement amplification (SDA) (Chen et al., 2018a; Tang et al., 2020), helicase-dependent amplification (HDA) (Moura-Melo et al., 2015; Liu et al., 2020), and catalyzed hairpin assembly (CHA) (Chen et al., 2018b; Chai et al., 2019), have also been applied to electrochemical detection of biological analytes. Recent papers have mainly focused on detection of miRNA. For example, toehold-mediated SDA was combined with surface-enhanced Ramen-scattering electrochemistry for dual-mode detection of miRNA, achieving an LOD as low as 0.12 fM (Zhou et al., 2021). We cite relevant reviews on isothermal amplification for readers interested in further reading (Monis and Giglio, 2006; Zhao et al., 2015).

In conclusion, target-based amplifications strategies can be applied towards a variety of targets. One-pot isothermal methods and enzyme-free amplification strategies are particularly appealing for POC settings, as they significantly decrease resource requirements. Furthermore, with the advent of increasingly sensitive and effective isothermal amplification methods, there is ample space to develop electroactive sensors for infectious diseases.

## Signal-Based Amplification Strategies

We classify signal-based amplification methods as methods that lower the LOD by modifying the transducer system to improve the signal-to-noise ratio. While target-based amplification commonly requires nucleic acids to aid in the detection of non-nucleic acid targets, signal-based amplification is generally accomplished through the engineering of abiotic sensor components. We identify recent advances in the detection of nucleic acids and whole pathogens based on two approaches: increasing the transducer surface area and increasing the detectable signal, as shown in Figure 2.

One prevalent strategy is to increase the electroactive surface area, especially through the functionalization of an electrode surface with conductive nanoparticles. For additional information on the subject, we point to reviews on nanoparticle-based electrochemical sensors (Cao et al., 2011; Ding et al., 2013; Lim and Ahmed, 2016). We focus specifically on gold nanoparticles (Khater et al., 2019; Lv et al., 2019; Cajigas et al., 2020) due to their conductivity, biocompatibility, and ease of deposition (Wang et al., 2014; Bo et al., 2018). In one example, Layqah et al. developed an electrochemical immunosensor for Middle East respiratory syndrome coronavirus (MERS-CoV) (Layqah and Eissa, 2019). The assay was performed on an array of carbon electrodes modified with gold nanoparticles and antibodies. Binding of the virus to the immobilized antibodies blocks access of the ferro/ferricyanide redox couple to the electrode surface, leading to a decrease in the current. The final sensor achieved an LOD of 1.0 pg/ml for MERS-CoV as well as selectivity over other respiratory viruses, such as influenza A and B. The gold nanoparticles increased the electroactive surface area and electrode surface loading, leading to an improvement in signal.

Nanostructuring of the electrode surface can similarly increase the active surface area on which biorecognition elements can be immobilize without overcrowding the individual binding sites, thereby increasing the sensitivity (Soleymani et al., 2011; Das et al., 2012; Das and Kelley, 2013). For example, Yousefi et al. developed a reagent-free sensor for the detection of SARS-CoV-2 that is ideal for on-demand testing in low-resource settings (Yousefi et al., 2021). The electrodes were fabricated from silicon wafers and coated with two layers of nanostructured gold, then functionalized with antibodies modified with electroactive ferrocene tags. Upon binding, the hydrodynamic drag of the virion-antibody complex increases, inducing a slower current decay. Critical to this method is the nanostructuring of the electrode, which both allows for higher loading density of the antibodies and more spacing between the antibodies, reducing the binding steric hindrance.

The second signal-based amplification approach is to increase the amount of detectable signal. As nanoparticles have a high surface area-to-volume ratio, they can be modified with more electroactive labels, thus increasing the detectable signal and improving the LOD of sensors. Zhao et al. describe the fabrication of an electrochemical sensor for the detection of SARS-CoV-2 RNA with a reported LOD of 200 copies/ml (Zhao et al., 2021). This sensor utilizes a “supersandwich-type” recognition strategy, in which the target sequence is simultaneously captured on an electrode and labeled with gold nanoparticle-toluidine blue (TB) complexes. The nanoparticles yield multiple labels of electroactive TB per RNA, thereby increasing the signal. The electrochemical detection was conducted with a smartphone, which is ideal for a POC field-deployable setting. We note that that the electroactive label does not necessarily have to be loaded on nanocomposites; aptamers and antibodies can also be used (Chen et al., 2006; Hosseini Ghalehno et al., 2019; Xiong et al., 2021).

Nanostructuring can also amplify the signal for impedance-based detection (Dahlin et al., 2012). Impedance-based sensors are uniquely suitable for the monitoring of bacterial pathogens due to the sensitivity of this technique, label-free operation, and high reproducibility (Furst and Francis, 2019). Moakhar et al. developed a plasmonic-assisted impedimetric sensor for multiple species of pathogenic bacteria based on hybrid structures of 3D gold nano-/micro-islands (Siavash Moakhar et al., 2020). The nanostructured gold surface has hierarchical 3D islands of gold that, when combined with graphene and illuminated with light, demonstrates plasmonic qualities that significantly increased the signal from impedance spectroscopy. The LOD of the system is 20 CFU/ml with a dynamic range of 2 × 10 to 1 × 10^5^ for *E. coli* and 1 × 10^2^ to 1 × 10^5^ CFU/ml for *S. epidermis*. This strategy is particularly appealing, as it is a label and reagent-free direct method.

Another label-free method for amplifying the detectable signal is the addition of electroactive cations in the detection buffer. Hexaammineruthenium (RuHex) is a positively charged redox-active metal complex that is used as a nucleic acid reports by interacting stoichiometrically with the negatively charged backbone. The RuHex signal can be further amplified with the addition of electroactive ions, such as ferricyanide, to improve the turnover of electrochemical reduction. Ferricyanide re-oxidizes the reduced Ru^2+^, increasing redox mediator turnover and significantly increasing the signal (Lapierre et al., 2003). This strategy has been utilized by Das et al. to detect clinically relevant mutations in a tumor growth-associated gene (Das et al., 2018). We note that this strategy has been applied for the sensing of ultralow concentrations of nucleic acids such as miRNA, but has not yet been used for infectious diseases (Islam et al., 2018; Wang et al., 2019; Das and Kelley, 2020). This is a viable strategy to increase the current output for the detection of viral or bacterial genetic material, and is especially useful for the design of POC sensors due to its ease of operation and minimal storage requirements.

## Perspective and Discussion

Electrochemical target-based amplification techniques can greatly improve the sensitivity and selectivity of electrochemical biosensors, while reducing the cost and operation time for pre-detection labeling and reliance on expensive instrumentation. While these strategies can significantly improve the LOD, they can only be applied to nucleic acids, limiting their application range. Furthermore, while recent amplification strategies have utilized one-pot isothermal applications that are more conducive to POC diagnostics, many still rely on the use of specialized reagents that are expensive and difficult to store. For example, LAMP requires enzymes and the design of specialized primers, and it is sensitive to off-target amplification and contamination; RCA requires padlock-shaped nucleic acid probes that are approximately 100 bps, driving up the synthesis cost (Zhang et al., 2021). Finally, clinical sample matrices often contain interfering biomolecules that reduce the efficacy of amplification enzymes, often necessitating additional purification steps. Enzyme-free methods such as HCR mitigate this disadvantage, making it a more appealing option.

Compared to target-based amplification, signal-based amplification has several notable advantages. The machinery to improve the signal is pre-built into the electrode, which also reduces the reagents and instrumentation needed during the sensing procedure. This is appealing for field-deployable applications, as they can be utilized by untrained personnel. Furthermore, these methods avoid off-target amplification, contamination, and the need for expensive and difficult-to-store enzymes. However, out of the examples introduced in this review, many require the use of nanocomposites or nanostructuring of the electrode, which require synthesis with expensive reagents, fabrication in cleanrooms, and specialized instrumentation. This adds an additional hurdle for the operation of POC sensors in low-resource settings. We also note that label-free impedimetric sensors require specially constructed handheld potentiostats that cost an order of magnitude more than amperometric potentiostats.

We also observe that many sensors use multiple signal amplification techniques or utilize combinations of materials to further drive down the LOD of the system. While these strategies can increase the sensitivity and specificity of detection, each additional method adds a layer of complexity to the synthesis, reagent requirements, and operation procedure, which can drive up the cost-per-test and time to result.

Despite the advances in sensing strategies, target range, and LOD, few electrochemical sensors have achieved widespread commercial use like the glucose sensor (Kissinger, 2005). Complicated sensor preparation protocols, cold-chain storage requirements, and complex operational steps limit the application of electrochemical biosensors. In order to improve their practical applicability, we argue that amplification strategies should have different levels of complexity based on the proposed usage. POC electrochemical biosensors can be further divided into two categories: a) for high resource, laboratory/clinical settings, and b) for low-resource, field-deployable, or personal-use settings. In laboratory or clinical settings where trained operators have access to cold storage and costly instrumentation, target-based amplification sensors with low LODs and large ranges of detection are preferred. Combinations of amplification methods with more intricate operation are further supported, and sensors can also be designed to support multiplexed sensing.

In comparison, sensors designed to be deployed in low-resource, field-deployable settings, or for personal use should require minimal reagents and storage specifications. Rather than simply minimizing the LOD, the sensor should be designed to either a) detect the minimal infectious dose of the target disease, or b) report a qualitative, rather than quantitative, result. Another critical design parameter should be its cost-per-test. We suggest that cost analysis be conducted on sensors for low-resource settings. Finally, the ease of operation should also be assessed. POC sensor design should focus on utilizing reagents that can be stored long-term at room temperature and operated by untrained personnel. Signal-based amplification strategies that utilize electroactive ions as amplifiers or pre-fabricated low-cost electrodes with long term stability are better for these settings. In conclusion, signal amplification techniques will enable electrochemical POC devices to be deployed for a wide variety of illnesses and settings, but should be designed with the application and target disease in mind.

## Data Availability

The original contributions presented in the study are included in the article; further inquiries can be directed to the corresponding author.
